# RNA Splicing: A Versatile Regulatory Mechanism in Pediatric Liver Diseases

**DOI:** 10.3389/fmolb.2021.725308

**Published:** 2021-09-28

**Authors:** Jian-Li Zhou, Yu-Zhen Zhao, Shan-Shan Wang, Mo-Xian Chen, Shaoming Zhou, Chen Chen

**Affiliations:** ^1^ Division of Gastroenterology, Shenzhen Children’s Hospital, Shenzhen, China; ^2^ School of Life Sciences and Biopharmaceutics, Guangdong Pharmaceutical University, Guangzhou, China; ^3^ Co-Innovation Center for Sustainable Forestry in Southern China, College of Biology and the Environment, Nanjing Forestry University, Nanjing, China; ^4^ Department of Infectious Disease, Nanjing Second Hospital, Nanjing University of Chinese Medicine, Nanjing, China

**Keywords:** alternative splicing, children, liver disease, non-alcoholic fatty liver disease, RNA sequencing, splicing-related protein

## Abstract

With the development of high-throughput sequencing technology, the posttranscriptional mechanism of alternative splicing is becoming better understood. From decades of studies, alternative splicing has been shown to occur in multiple tissues, including the brain, heart, testis, skeletal muscle, and liver*.* This regulatory mechanism plays an important role in physiological functions in most liver diseases. Currently, due to the absence of symptoms, chronic pediatric liver diseases have a significant impact on public health. Furthermore, the progression of the disease is accelerated in children, leading to severe damage to their liver tissue if no precautions are taken. To this end, this review article summarizes the current knowledge of alternative splicing in pediatric liver diseases, paying special attention to liver damage in the child stage. The discussion of the regulatory role of splicing in liver diseases and its potential as a new therapeutic target is also included.

## Introduction

Chronic liver disease is an increasing health burden in children and adults. Although the incidence is unclear, it is estimated that chronic liver disease has become the 11th leading cause of death among adults (Vos et al., 2016; Gao et al., 2021). In addition, many children are hospitalized due to liver diseases each year. In children and adults, nonalcoholic fatty liver disease (NAFLD) is known as a frequently appearing chronic liver disease with a complex interplay between environmental and genetic factors in industrialized countries (Vos et al., 2016; Gao et al., 2021). It is generally accepted that pediatric liver diseases, including NAFLD, chronic viral hepatitis, and other chronic liver diseases, may develop into chronic diseases, such as cirrhosis and hepatocellular carcinoma, in adults (Della Corte et al., 2016; Nobili et al., 2019). In particular, the number of liver cancer cases has increased rapidly since 1990 (Della Corte et al., 2016). In addition to liver-associated symptoms, NAFLD is related to a risk increment of diabetes (type 2) and cardiovascular disease in adults (Draijer et al., 2019). Furthermore, hepatic cholestasis and metabolic and autoimmune liver problems have been demonstrated to be the most common causes of liver failure in children, leading to liver transplantation at their final stage (Nikeghbalian et al., 2021). On the other hand, viral hepatitis, alcoholic hepatitis, and hepatocellular carcinoma are more common in adults. In both children and adults, the vast majority of patients are asymptomatic in the early stage of disease, and the prevalence of these diseases is unknown, leading to delays in diagnosis and treatment. Furthermore, because similar responses will be shown from different injuries of hepatic cells, different types of liver diseases may show similar presentations among children (Della Corte et al., 2016). Moreover, nonspecific signs, including abdominal pain, fatigue, loss of appetite, pruritus, or hepatomegaly, can also be present among patients. Thus, prevention and early diagnosis are important to distinguish these chronic liver diseases and will lead to cost-effective treatments in pediatric settings.

Alternative splicing is the process to mediate pre-messenger RNA maturation into mRNA, by removing introns and reattaching exons (Webster, 2017). During this precise molecular process, different transcripts are assembled with the help of splice sites and associated sequences. Hence, alterations in splicing factors, such as the usage of new splice sites or enhancer sequences, as well as disruption of splicing sequences, can lead to diseases including metabolic diseases, liver diseases, cancers, and neurodegenerative diseases (Montes et al., 2019; Rahman et al., 2020). For instance, mutations in spliceosome RNA genes were detected in hepatocellular carcinoma, medulloblastoma, and chronic lymphocytic leukemia (Jimenez et al., 2018; Suzuki et al., 2019; Rahman et al., 2020). In addition, critical changes were found in the function of RNA splicing in hepatic fat metabolism of obesity (Pihlajamaki et al., 2011). Recent studies have also demonstrated alterations in the splicing machinery in inflammation, steatosis, and fibrosis in NAFLD patients and animals (Zhu et al., 2016; Gerhard et al., 2018; Del Río-Moreno et al., 2019; Hoang et al., 2019; Wang et al., 2019). In this review article, we will summarize the appearance of alternative RNA splicing in pediatric liver diseases and highlight its roles in the development and progression of these diseases (Table 1).

**TABLE 1:** Studies reporting alterations of RNA splicing in pediatric liver diseases.

Study objects	Methods	References
Splicing mutations in ATP8B1 of PFIC	The effect of premessenger RNA splicing on 14 ATP8B1 exon-intron boundary mutations was studied using an *in vitro* microgene system	van der Woerd et al. (2015)
Splicing variant in JAG1	The candidate variants were verified by Sanger sequencing, and the splicing effect of the candidate variants was clarified by RNA detection	Chen et al. (2020)
The FXR splicing through transcriptional program in NAFLD	The FXR variant gene was transferred into the liver of FXR (−/−) mice to evaluate its effect *in vivo*	Correia et al. (2015)
Transcriptomic analysis in NAFLD	Complete transcriptome analysis of intraperitoneal adipose tissue (IAT) in severely obese adolescents was performed using RNA sequencing	Sheldon et al. (2016)
Alternative splicing of hepatitis B virus	The regulation of splicing of HBV in chemically and surgically induced liver injury was studied in transgenic mice with whole HBV genomes and hepatocellular carcinoma cells	Duriez et al. (2017)
Alternative splicing of AZIN1 in hepatitis C virus	Seven splicing variants of AZIN1 (SV2-8) were cloned from human hepatic stellate cell line LX2 by polymerase chain reaction	Paris et al. (2011)
The spliceosome factor SART1 in HCV	SiRNA knockout and mRNA sequencing in Huh7.5.1 cells selection genes for mRNA variation and their proteins, and HCV replication	Lin et al. (2015)
Transcriptome Analysis in Pediatric Hepatocellular Carcinoma	The activity of YAP and the expression of Hippo pathway components in tumor and non-tumor liver tissues of 7 children with HCC were detected	Laquaglia et al. (2016)
Transcriptome profiling of biliary atresia	Liver samples from infants with biliary atresia were collected and transcriptome analysis was performed using RNA-seq technique	Xiao et al. (2014)
Long noncoding RNA H19 (lncRNAH19) in biliary atresia	Liver specimens from 53 BA patients and 11 control liver specimens were analyzed by qRT-PCR, Western blotting, histology, and immunohistochemistry (IHC)	Xiao et al. (2019)
Transcriptomic of human hepatocellular carcinomas and hepatoblastomas	The gene expression patterns and global genomic changes of HCC and HBS were analyzed	Luo et al. (2006)

## Common Liver Diseases in Children and Adults and Their Relationship With RNA Splicing

### Splicing Mutations Identified in Infantile Cholestasis

Infantile cholestasis (IC) is an impairment of bile production or flow occurring in the first months of life that affects 1:2,500 live births (Pietrobattista et al., 2020). It is recognized as an important cause of chronic liver disease in infants and young children, including biliary atresia (BA) (35–41%), progressive familial intrahepatic cholestasis (PFIC) (10%), Alagille syndrome (2–6%), and other causes (Götze et al., 2015). This paragraph will discuss the regulatory role of alternative splicing (AS) in PFIC and Alagille syndrome. The association between AS and BA will be stated later. PFIC patients diagnosed in childhood with intrahepatic cholestasis frequently progress to end-stage liver disease before adulthood (Bull and Thompson, 2018). There are three major proteins affected in PFIC, including the bile salt export pump (BSEP) encoded by *ABCB11*, multidrug resistance protein 3 (MDR3) encoded by *ABCB4*, and a membrane lipid composition protein (FIC1) encoded by *ATP8B1*, while three other reported proteins may be affected in PFIC patients, including tight junction protein 2 encoded by *TJP2*, the farnesoid X receptor (FXR) encoded by *NR1H4*, and myosin 5B encoded by *MYO5B* (Sambrotta et al., 2014; Qiu et al., 2017; Keitel et al., 2019). To date, except for *NR1H4*, splicing mutations have been found in other five genes associated with PFIC (http://www.hgmd.org) (Liu et al., 2010; van der Woerd et al., 2015; Khabou et al., 2016; Stenson et al., 2017; Al-Hussaini et al., 2021). In particular, FIC1 is part of the p-type adenosine triphosphatase type 4 subfamily involved in membrane phospholipid transport (Paulusma et al., 2008). This protein is found in the apical membrane of hepatocytes and is considered to be a phospholipid translocase that carries phospholipids, such as phosphatidylethanolamine (PE) and phosphatidylserine (PS), from the ectoplasmic lobule of the outer tubule to the cytoplasmic lobule of hepatocytes. FIC1 protects the tubule membrane by maintaining plasma membrane asymmetry (Vitale et al., 2019). Previous studies systematically described 14 mutations at the exon-intron boundary of *ATP8B1* and found that most of them caused aberrant splicing of its gene product (van der Woerd et al., 2015). Furthermore, this study suggested that compensatory modified U1 small nuclear RNAs (snRNA), which are complementary to the mutated donor splicing site, are highly effective in improving exon definition, implying the therapeutic potential of these mutated loci.

Furthermore, BSEP is a binding cassette transporter for adenosine triphosphate and is involved in the transfer of bile salts from hepatocytes to bile ducts, a process which is essential for maintaining the enterohepatic circulation of related bile salts (Vitale et al., 2019). Mutations in BSEP disrupt the transportation process of bile salts out of hepatocytes, resulting in increased concentrations of intracellular bile salts to damage hepatocytes (Vitale et al., 2019). Moreover, MDR3 (*ABCB4*) encodes a phosphatidylcholine (PC) flippase with two transmembrane and cytoplasmic nucleotide binding domains, respectively. This protein is specifically localized at the tubular membrane of hepatocytes to mediate PC transportation from hepatocytes to bile ducts (Vitale et al., 2019). Over-expression of MDR3 increases the transport rate of fluorescin-labeled PC, but not other phospholipids. The abnormal function of MDR3 leads to the depletion of PC in the biliary tubules and elevated free hydrophobic bile acids in the space, causing damage to bile duct cells and the development of cholestasis (Henkel et al., 2019).

In addition, TJP2 is a tight junction protein that can interact with actin cytoskeletons and liver-specific tight junction proteins, such as CLDN1 and CLDN2 (Sambrotta and Thompson, 2015; González-Mariscal et al., 2019). Tight junctions are able to prevent biliary elements from leaking into the liver parenchyma. However, upon TJP2 mutation, CLDN1 failed to localize to its original position in the hepatic lobular parenchyma. This mis-localization disrupts the tight junctions, causing the leakage of bile salts with cytotoxicity into the paracellular space and resulting in damage to bile duct cells and surrounding hepatocytes (Sambrotta et al., 2014; Sambrotta and Thompson, 2015). Previous studies have reported that the mutant C. 2180-5T>G caused the jump of exon 15 of TJP2 and the deletion of 32 amino acid residues in the framework (Zhang et al., 2020). Thus, this mutation can serve as potential diagnostic targets of this disease.

At last, the fifth protein is known as myosin 5B (MYO5B), identified as a molecular motor associated with actin (Vitale et al., 2019). Reports have indicated that MYO5B can interact with RAS-associated GTP-binding protein 11A (RAB11A), in order to assist the polarization process of epithelial cells. Meanwhile, the localization of BSEP at the tubule membrane is affected by the activity of this interaction (Girard et al., 2014; Overeem et al., 2020). Mutations in this gene have been linked to microvillus inclusion body disease (MVID), which affects enterocytes, leading to reducing bile acid uptake, diarrhea, malabsorption (Van IJzendoorn et al., 2020; Overeem et al., 2020). Qiu et al. reported that three classical splicing mutations are identified from their study and this type of mutation is defined as severe mutations. Taking into consideration the interaction between MYO5B and RAB11A, the manipulation of this upstream signal transduction module may provide additional targets for therapeutic purposes. As a congenital disorder, Alagille syndrome is characterized by eye and heart abnormalities, skeletal deformities, cholestasis, and characteristic facial features (Li et al., 1997). Nearly 94% of patients have congenital cardiac diseases, and 21–31% of patients may be candidates for liver transplantation (Li et al., 1997). Currently, approximately 94% of Alagille syndrome patients have variants of *JAG1*, while 1–2% of patients have *NOTCH2* (McDaniell et al., 2006). Both JAG1 and NOTCH2 are identified as single-channel transmembrane proteins, containing 26 and 34 exons, respectively. Specifically, the interaction between ligand JAG1 and NOTCH2 receptor requires several functional motifs, such as the C2-like domain, delta-Serate-lag2 (DSL) domain, epidermal growth factor-like (EGF-like) repeats of *JAG1,* and extracellular EGF-like repeats on *NOTCH2* (Chillakuri et al., 2013; Kopan and Ilagan, 2009; Lindsell et al., 1995), and the mutation of this pathway identified in children with Alagille syndrome. In addition, only one *NOTCH2* mutation of the splice site of exon 33 (c.5930−1G→A) was identified in the patient with Alagille syndrome, while more than 40 splicing mutations were reported in the *JAG1* gene with Alagille syndrome (http://www.hgmd.cf.ac.uk/ac/gene.php?gene=JAG1) (Mcdaniell et al., 2006; Chen et al., 2018). However, how these mutations affect the interaction of this pathway remains to be elucidated. Nevertheless, these reported splicing mutations may serve as potential diagnostic targets for Alagille syndrome.

### Transcriptome Studies of RNA Splicing in Metabolic Liver Diseases

Metabolic liver diseases might be the second leading cause of liver transplantation in children, including NAFLD, Wilson’s disease (WD), alpha-1 antitrypsin, and glycogen storage disease (Elisofon et al., 2020). NAFLD is the most common chronic liver disease in children and adolescents worldwide, notified to be the second most common cause of liver transplantation (Goldner and Lavine, 2020). Approximately, 2.6–11.3% of children and approximately 40–70% of obese children are diagnosed with NAFLD worldwide (Goldner and Lavine, 2020). Consequently, NAFLD affects public health, especially children and adolescents.

Several genetic variants, including genes encoding transmembrane 6 superfamily member 2 (*TM6SF2*), patatin-like phospholipase domain-containing 3 (*PNPLA3*), glucokinase regulator (*GCKR*), and membrane bound O-acyl transferase 7 (*MBOAT7*), contribute to the risk of NAFLD, whereas protein phosphatase 1 regulatory subunit 3B (*PPP1R3B*) has been documented to have a protective effect against NAFLD (Valenti et al., 2010; Santoro et al., 2012; Kozlitina et al., 2014; Mancina et al., 2016; Dongiovanni et al., 2018; Li et al., 2020). However, other genetic variants, such as Mer tyrosine kinase (*MERTK*), interferon-λ4 (*IFNL4*), and 17-β hydroxysteroid dehydrogenase 13 (*HSD17B13*), might modify the fibrotic effect of NAFLD, which were highlighted as new candidate genes among Hispanic boys (Petta et al., 2016; Petta et al., 2017; Wattacheril et al., 2017; Abul-Husn et al., 2018). Furthermore, the hepatic expression of farnesoid X receptor (FXR) was reduced in both animal models and NAFLD patients, where hepatic FXR expression was reduced in nonalcoholic steatohepatitis (NASH) (Yang et al., 2010). A study showed that FXR splicing toward FXRα2 reduced hepatic lipid accumulation through the transcriptional program, which could greatly enhance the therapeutic effect by improving pharmacological targeting of select FXR agonists (Correia et al., 2015). This evidence suggests that FXR agonists could be a potential therapy for NAFLD. Moreover, the intra-abdominal adipose tissue (IAT) of severely obese adolescents with NAFLD has unique transcriptome differences, providing important molecular markers for identifying potential therapeutic targets for childhood NASH (Sheldon et al., 2016). In a previous study, reduced fatty acid desaturase 1 (FADS1) function was related to NAFLD and responded to treatment in children through FADS1 transcription levels (Nobili et al., 2018). In short, the study between RNA splicing and NAFLD in children was often conducted at the transcriptome level, while there was much more documented evidence about alternative RNA splicing in NAFLD in adults (Wu et al., 2021).

WD is characterized by a series of hepatic, neurological, and psychiatric symptoms, which result from impaired copper excretion at the bile location. It is an autosomal recessive disorder caused by a mutation in the *ATP7B* gene (Shah et al., 1997). Genetic prevalence is 3–4 times higher than clinical estimates, although the initial prevalence of 1:30,000–1:50,000 remains valid in at least Asia, the United States, and Europe (Sandahl et al., 2020). More than 70 splicing mutations have been reported at this genetic locus worldwide, including exon skipping and acceptor and donor splice site mutations (http://www.hgmd.cf.ac.uk/ac/gene.php?gene=ATP7B) (Lovicu et al., 2009; Zappu et al., 2012; Mameli et al., 2015; Stenson et al., 2017; Wang et al., 2018), suggesting that this locus is a hotspot of splicing mutation. Moreover, those splicing mutations of the *ATP7B* gene can be used as the diagnostic targets for WD.

### Splicing Regulation in Viral Hepatitis

Hepatitis B virus (HBV) and hepatitis C virus (HCV) are responsible for a major burden of viral hepatitis worldwide. The prevalence of chronic hepatitis B infection in children has been reduced due to improved hygiene measures, blood supply, and introduction of universal vaccination for this virus (Della Corte et al., 2016). AS regulation of HBV transcripts has been reported *in vitro* and in the liver of HBV-infected patients (Suzuki et al., 1989; Wu et al., 1991). In particular, AS could regulate the splicing of 3.5 KB HBV pregenomic RNA (pGRNA), which encodes either capsid or polymerase proteins to facilitate viral genome replication (Seeger and Mason, 2015).

The main HBV pGRNA splicing variant, single presplicing genomic RNA (SP1RNA), harbors a deletion of 1/3 of the viral genome and accounts for approximately 30% of the total HBV pGRNA in hosts, suggesting the importance of AS regulation in virus packaging, reverse transcription, and virus release (Terré et al., 1991; Soussan et al., 2008; Bayliss et al., 2013). In particular, host splicing factors, including SF1, hnRNPAB, PSF, LA, and some SRSFs, have been demonstrated to participate in the splicing regulation of HBV pGRNA (Soussan et al., 2008).

With the accurate screening of blood products and organ donors, the prevalence of hepatitis C infection has been significantly reduced, and vertical transmission is the main source of infection (Della Corte et al., 2016). A single nucleotide polymorphism (SNP) variant in the antizyme-inhibitor-1 (*AZIN1*) gene called *AZIN1* SV2 (*AZIN1* splice variant 2) leads to a novel alternative spliced isoform that modifies the fibrogenic potential of hepatic stellate cells (HSCs) in HCV cirrhotic livers (Huang et al., 2007; Paris et al., 2011). Previous research indicated that the spliceosome factor SART1 (squamous cell carcinoma antigen recognized by T cells) regulates HCV replication by altering its expression and splicing level (Lin et al., 2015).

### Pediatric Liver Tumors

Malignant liver tumors are rare in children, accounting for only 1% of all malignancies (Spector and Birch, 2012). While more than two-thirds of them are hepatoblastomas (HBs), 20% are hepatocellular carcinomas (HCCs). The latter section will discuss the relationship between AS and HB. Here, we only discuss AS in HCC, which is typically in older children or adolescents and is the major type of adult liver cancer (Crippa et al., 2017). At present, hepatocyte proliferation and HCC development are closely related to the transcriptional coactivator Yes-associated protein and its targeted Hippo pathway in animal models (Dong et al., 2007). Previous studies have shown that the mRNA expression of Yes-associated protein (YAP) target genes (*CCNE1*, *CTGF*, *Cyr61*) was increased in pediatric HCC, demonstrating an enrichment of YAP nuclear localization and its activity in moderately differentiated pediatric HCC (LaQuaglia et al., 2016). However, the relationship between AS and HCC in children has rarely been reported in comparison to adult patients.

## Liver Diseases Uniquely Present in Children and Their Splicing Regulation

### Post-Transcriptional Regulation in Biliary Atresia

BA is caused by bile duct occlusion or interruption from the hilum to the duodenum and is becoming the most common cholestatic liver disease leading to pediatric liver transplantation. The incidence of BA ranges from 1 in 5,000 cases to 1 in 19,000 cases, with higher rates in Asia than in European countries (Fawaz et al., 2017). A number of likely causal proteins of BA have been identified in previous studies, including *FOXA2* (Tsai et al., 2015), *CFC1* (Davit-Spraul et al., 2008), *ZEB2* (Cui et al., 2011), *ZIC3* (Ware et al., 2004), *HNF1B* (Shaalan et al., 2019), *PKD1 L1* (Berauer et al., 2019), *GPC1* (Cui et al., 2011), *XPNPEP1* (Garcia-Barceló et al., 2010), *ADD3* (Tsai et al., 2014), *EFEMP1* (Chen et al., 2018), *ARF6* (Ningappa et al., 2015), *STIP1*, and *REV1* (Rajagopalan et al., 2020), without splicing mutations (Table 2). However, no genes have been identified as a definite cause of isolated BA cases so far (Girard and Panasyuk, 2019). Nevertheless, the top 10 upregulated loci identified by transcriptome approach from BA samples, including *CSRNP1*, *IL6R*, *CPB2*, *TTR*, *TD O 2*, *SERPINC1*, *C6*, *DHTKD1*, *IGFBP1*, and *RDH16,* might deepen our understanding of the transcriptional and post-transcriptional mechanisms among BA patients (Xiao et al., 2014).

**TABLE 2:** Genetic manipulation of RNA splicing in pediatric liver disease.

Gene	Related diseases	Splicing mutation	Potential function	RNA splicing or genetic variants	As potential diagnostic/therapeutic targets	References
*ATP8B1/FIC1*	Progressive Familial Intrahepatic Cholestasis type 1	14 splicing mutations	exon skipping, donor, acceptor, splice sites	Splicing mutations associated with PFIC	As novel therapeutic targets	van der Woerd et al. (2015)
*ABCB11/BESP*	Progressive Familial Intrahepatic Cholestasis type 2	c.784 + 1G > C	Splice sites	Splicing mutations associated with PFIC	As diagnostic targets	Sharma et al. (2018)
*ABCB4/MDR3*	Progressive Familial Intrahepatic Cholestasis type 3	c.3487–16T>C; c.175C>T	Without mention	Splicing mutations associated with PFIC	As diagnostic targets	Khabou et al. (2016)
*TJP2*	Progressive Familial Intrahepatic Cholestasis type 4	c.2180–5T>G	Exon skipping	Splicing mutations associated with PFIC	As diagnostic targets	Zhang et al. (2020)
*MY O 5B*	Progressive Familial Intrahepatic Cholestasis type 5	c.3538-1G>A; c.2414+5G>T; c.4852 + 11A>G	Splice sites	Splicing mutations associated with PFIC	As novel therapeutic targets	Qiu et al. (2017)
*JAG1*	Alagille syndrome	49 splicing mutations include c.2917–8C > A	Exon skipping, donor, acceptor, splice sites	Splicing mutations associated with Alagille syndrome	As diagnostic targets	Chen et al. (2020); (http://www.hgmd.cf.ac.uk/ac/gene.php?gene=JAG1)
*NOTCH2*	Alagille syndrome	c.5930-1G→A	Splice acceptor	Splicing mutations associated with Alagille syndrome	As diagnostic targets	Mcdaniell et al. (2006)
*PNPLA3*	Nonalcoholic fatty liver disease	Without mention	Without mention	Genetic variants associated with NAFLD	Uncertainty	Valenti et al. (2010)
*TM6SF2*	Nonalcoholic fatty liver disease	Without mention	Without mention	Genetic variants associated with NAFLD	Uncertainty	Kozlitina et al. (2014)
*GCKR*	Nonalcoholic fatty liver disease	Without mention	Without mention	Genetic variants associated with NAFLD	Uncertainty	Santoro et al. (2012)
*MBOAT7*	Nonalcoholic fatty liver disease	Without mention	Without mention	Genetic variants associated with NAFLD	Uncertainty	Mancina et al. (2016)
*MERTK*	Nonalcoholic fatty liver disease	Without mention	Without mention	Genetic variants associated with NAFLD	Uncertainty	Dongiovanni et al. (2018)
*IFNL4*	Nonalcoholic fatty liver disease	Without mention	Without mention	Genetic variants associated with NAFLD	Uncertainty	Petta et al. (2017)
*HSD17B13*	Nonalcoholic fatty liver disease	Without mention	Without mention	Genetic variants associated with NAFLD	Uncertainty	Abul-Husn et al. (2018)
*FXR*	Nonalcoholic fatty liver disease	FXR splicing toward FXRα2 reduced hepatic lipid accumulation through the transcriptional program	Enhance the therapeutic effect by improving pharmacological targeting of select FXR agonists	RNA splicing associated with NAFLD	As therapeutic targets	Correia et al. (2015); Sheldon et al. (2016)
*ATP7B*	Wilson’s disease	71 splicing mutations include c.51 + 4 A → T; c. 2,121 + 3 A → G; c.2447 + 5 G → A; 1946 + 6 T→C; c.52–2,671_368del3039	Exon skipping, acceptor, and donor splice	Exon skipping, acceptor, and donor splice site associated with WD	As diagnostic targets	Lovicu et al. (2009); Zappu et al. (2012); Mameli et al. (2015); Wang et al. (2018); (http://www.hgmd.cf.ac.uk/ac/gene.php?gene=ATP7B)
*CCNE1*	Hepatocellular carcinomas	Without mention	mRNA expression was increased in the pediatric HCC	YAP target gene mRNA expression was increased in the pediatric HCC	Uncertainty	Laquaglia et al. (2016)
*CTGF*	Hepatocellular carcinomas	Without mention	mRNA expression was increased in the pediatric HCC	YAP target gene mRNA expression was increased in the pediatric HCC	Uncertainty	Laquaglia et al. (2016)
*Cyr61*	Hepatocellular carcinomas	Without mention	mRNA expression was increased in the pediatric HCC	YAP target gene mRNA expression was increased in the pediatric HCC	Uncertainty	Laquaglia et al. (2016)
*FOXA2*	Biliary atresia	Without mention	Without mention	Likely causal genes of BA	Uncertainty	Tsai et al. (2015)
*CFC1*	Biliary atresia	Without mention	Without mention	Likely causal genes of BA	Uncertainty	Davit-Spraul et al. (2008)
*ZEB2*	Biliary atresia	Without mention	Without mention	Likely causal genes of BA	Uncertainty	Cui et al. (2011)
*ZIC3*	Biliary atresia	Without mention	Without mention	Likely causal genes of BA	Uncertainty	Ware et al. (2004)
*HNF1B*	Biliary atresia	Without mention	Without mention	Likely causal genes of BA	Uncertainty	Shaalan et al. (2019)
*PKD1L1*	Biliary atresia	Without mention	Without mention	Likely causal genes of BA	Uncertainty	Berauer et al. (2019)
*GPC1*	Biliary atresia	Without mention	Without mention	Likely causal genes of BA	Uncertainty	Cui et al. (2013)
*XPNPEP1*	Biliary atresia	Without mention	Without mention	Likely causal genes of BA	Uncertainty	Garcia-Barceló et al. (2010)
*ADD3*	Biliary atresia	Without mention	Without mention	Likely causal genes of BA	Uncertainty	Tsai et al. (2014)
*EFEMP1*	Biliary atresia	Without mention	Without mention	Likely causal genes of BA	Uncertainty	Chen et al. (2018)
*ARF6*	Biliary atresia	Without mention	Without mention	Likely causal genes of BA	Uncertainty	Ningappa et al. (2015)
*STIP1*	Biliary atresia	Without mention	Without mention	Likely causal genes of BA	Uncertainty	Rajagopalan et al. (2020)
*REV1*	Biliary atresia	Without mention	Without mention	Likely causal genes of BA	Uncertainty	Rajagopalan et al. (2020)
*lncRNAH19*	Biliary atresia	Without mention	Suggesting its crucial role in BA bile duct cell proliferation and cholestatic liver injury	It was proposed to have a positive correlation with the BA-related severity of liver fibrosis	A valuable target for early diagnosis and the development of novel therapeutic procedures	Xiao et al. (2019)
*CTNNB1*	Hepatoblastoma	Without mention	Without mention	Genetic variants associated with HB	As therapeutic targets	Crippa et al. (2017); Sha et al. (2019)

Recently, long noncoding RNAH19 (lncRNAH19) was proposed to have a positive correlation with the BA-related severity of liver fibrosis (Xiao et al., 2019). Moreover, H19, a molecular sponge of the microRNA let-7 family, activates its downstream target, high mobility group member AT-hook 2 (HMGA2), during biliary tract proliferation, suggesting its crucial role in BA bile duct cell proliferation and cholestatic liver injury. Thus, lncRNAH19 may emerge as a valuable target for early diagnosis and the development of novel therapeutic procedures for BA patients.

Furthermore, recent studies have shown that the abnormal expression of long noncoding RNA Alu-mediated p21 transcription regulator (APTR) in the liver of BA infants may be pivotal for liver fibrosis in these patients (Makhmudi et al., 2020). Moreover, potential determinants of prognosis in Kasai portal enterostomy (KPE), such as phosphoenolpyruvate carboxykinase (PCK1) and matrix metalloproteinase-7 (MMP7), were determined by RNA sequencing data (Ramachandran et al., 2019). In particular, the abundance of MMP7 was higher in patients with failed jaundice clearance after KPE and in patients with end-stage liver disease (ESLD) than in the control group. In contrast, successful KPE treatment could induce PCK1 expression, and the abundance of PCK1 in patients with uncleared jaundice after KPE was repressed. Therefore, the abundance of MMP7 and PCK1 could be used as indicators for KPE outcome prediction and disease progression for clinicians.

### Splicing Mutation in Hepatoblastoma

Hepatoblastoma (HB) is the most common pediatric liver tumor, which typically occurs before the age of three and can be congenital (Trobaugh-Lotrario et al., 2013). The incidence of HBs has increased due to the greater numbers of premature births and infants with birth weights lower than 1,500 *g* (Feng et al., 2019). A β-catenin-encoding protein, *CTNNB1*, is the most frequently mutated HB gene, accounting for 50–90% of diagnosed HB cases (Crippa et al., 2017). Several genes, such as *Spondin2*, *Edil3*, *Glypican 3*, *Osteopontin*, and *PEG10*, were highly elevated, whereas *Ficolin 3* was downregulated in human HCC and HB cases (Luo et al., 2006). However, several genes, including *IGF2*, *fibronectin*, *DLK1*, *TGFb1*, *MALAT1*, and *MIG6,* were overexpressed in HB *versus* HCC.

HB is genetically characterized by abnormal activation of the Wnt/β-catenin signaling pathway (Sha et al., 2019). Generally, extensive evidence has suggested that mutations in the β-catenin gene exon 3 are responsible for the activation of the Wnt/β-catenin signal transduction pathway in HB. Furthermore, the accumulation of β-catenin proteins resulted from increased translocations to the nucleus and cytoplasm and is positively correlated to cancer severity. Therefore, the abundance of β-catenin and target genes from its signaling pathway can be used as diagnostic and prognostic markers for pediatric liver tumors. In addition, several research groups have proposed the therapeutic effects of HB by specific inhibition of Wnt/β-catenin pathway, through a number of post-transcriptional measures such as short interfering miRNA, RNAs (siRNA), and bioactive small molecules. Hence, the Wnt/β-catenin signaling pathway is a valuable target for the development of therapeutic measures of HB (Koch et al., 1999; Takayasu et al., 2001; Koch et al., 2005; Cairo et al., 2008; Eichenmüller et al., 2014; Sumazin et al., 2017; Sha et al., 2019). However, HB has the lowest mutation burden among all known cancer types, and the genetic determinants of HB remain to be further investigated (Gröbner et al., 2018). Less than 5 mutations per hepatoblastoma were identified by studies using whole-exome sequencing, suggesting that this low mutation frequency of HB hindered the potential targets that are responsible for HB progression (Eichenmüller et al., 2014; Jia et al., 2014; Sumazin et al., 2017).

## Future Perspectives

**FIGURE 1:**
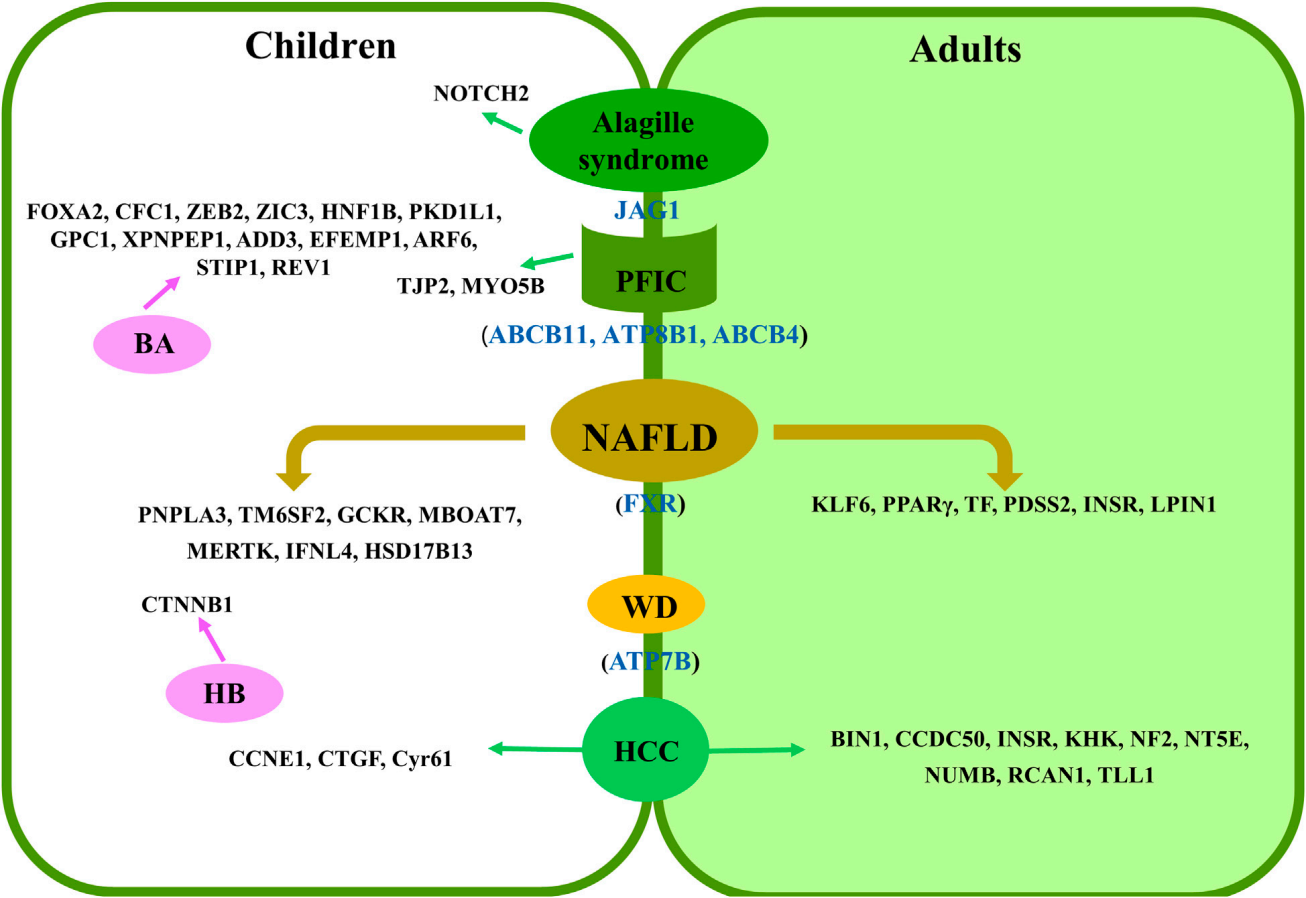
Summary of splicing-related loci in pediatric liver diseases. Shared loci by adults and children are presented as blue in color in brackets. Specific loci to children or adults are presented in the left and right panels, respectively.

There are many types of liver diseases in children, but many of them are rare in the world population. To date, much less research has been conducted on the association between RNA splicing and liver diseases in children than in adults (Figure 1). Furthermore, specific disease types at the child stage have also been reported to have splicing regulation on their potential genomic loci. In this review article, we found that there are many studies that performed their research on pediatric NAFLD in comparison to adult cases. This might be due to the longer life span of this disease at the child stage, which will greatly impact their life (Draijer et al., 2019). It has been expected that more targeted chemical drugs, such as FXR agonists, can be developed based on splicing variants to treat NAFLD. Although there have been no randomized controlled trials (RCTs) in children, this may be a major area for subsequent exploration (Jia et al., 2014). Intriguingly, most splicing mutations reported thus far lack functional studies at the molecular level, including those identified in PFIC, Alagille syndrome, and WD*.* Therefore, an in-depth study should be carried out to verify their roles in the corresponding diseases, evaluate the potential of these targets for drug development, and establish a noninvasive early diagnosis method. Specifically, these splicing events could be controlled by their upstream regulators, which have been demonstrated in adult and animal studies (Wu et al., 2021). Moreover, BA and HB, which occur in infancy or young children, seriously impact the health of children at this stage. Therefore, the molecular mechanism of these splicing variants in pediatric liver diseases requires further investigation.

## References

[B1] Abul-Husn N. S. Cheng X. Li A. H. Xin Y. Schurmann C. Stevis P. (2018). A Protein-TruncatingHSD17B13Variant and Protection from Chronic Liver Disease. N. Engl. J. Med. 378, 1096–1106. 10.1056/nejmoa1712191 29562163 PMC6668033

[B2] Al-Hussaini A. Lone K. Bashir M. S. Alrashidi S. Fagih M. Alanazi A. (2021). ATP8B1, ABCB11, and ABCB4 Genes Defects: Novel Mutations Associated with Cholestasis with Different Phenotypes and Outcomes. J. Pediatr. 236, 113–123. 10.1016/j.jpeds.2021.04.040 33915153

[B3] Bayliss J. Lim L. Thompson A. J. V. Desmond P. Angus P. Locarnini S. (2013). Hepatitis B Virus Splicing Is Enhanced Prior to Development of Hepatocellular Carcinoma. J. Hepatol. 59, 1022–1028. 10.1016/j.jhep.2013.06.018 23811301

[B4] Berauer J. P. Mezina A. I. Okou D. T. Sabo A. Muzny D. M. Gibbs R. A. (2019). Identification of Polycystic Kidney Disease 1 like 1 Gene Variants in Children with Biliary Atresia Splenic Malformation Syndrome. Hepatology 70, 899–910. 10.1002/hep.30515 30664273 PMC6642859

[B5] Bull L. N. Thompson R. J. (2018). Progressive Familial Intrahepatic Cholestasis. Clin. Liver Dis. 22, 657–669. 10.1016/j.cld.2018.06.003 30266155

[B6] Cairo S. Armengol C. De Reyniès A. Wei Y. Thomas E. Renard C.-A. (2008). Hepatic Stem-like Phenotype and Interplay of Wnt/β-Catenin and Myc Signaling in Aggressive Childhood Liver Cancer. Cancer Cell 14, 471–484. 10.1016/j.ccr.2008.11.002 19061838

[B7] Chen Y. Gilbert M. A. Grochowski C. M. Mceldrew D. Llewellyn J. Waisbourd-Zinman O. (2018). A Genome-wide Association Study Identifies a Susceptibility Locus for Biliary Atresia on 2p16.1 within the Gene EFEMP1. Plos Genet. 14, e1007532. 10.1371/journal.pgen.1007532 30102696 PMC6107291

[B8] Chen Y. Liu X. Chen S. Zhang J. Xu C. (2020). Targeted Sequencing and RNA Assay Reveal a Noncanonical JAG1 Splicing Variant Causing Alagille Syndrome. Front. Genet. 10, 1363. 10.3389/fgene.2019.01363 32038717 PMC6993058

[B9] Chillakuri C. R. Sheppard D. Ilagan M. X. G. Holt L. R. Abbott F. Liang S. (2013). Structural Analysis Uncovers Lipid-Binding Properties of Notch Ligands. Cel Rep. 5, 861–867. 10.1016/j.celrep.2013.10.029 PMC3888931 24239355

[B10] Correia J. C. Massart J. De Boer J. F. Porsmyr-Palmertz M. Martínez-Redondo V. Agudelo L. Z. (2015). Bioenergetic Cues Shift FXR Splicing towards FXRα2 to Modulate Hepatic Lipolysis and Fatty Acid Metabolism. Mol. Metab. 4, 891–902. 10.1016/j.molmet.2015.09.005 26909306 PMC4731735

[B11] Crippa S. Ancey P. B. Vazquez J. Angelino P. Rougemont A. L. Guettier C. (2017). Mutant CTNNB 1 and Histological Heterogeneity Define Metabolic Subtypes of Hepatoblastoma. EMBO Mol. Med. 9, 1589–1604. 10.15252/emmm.201707814 28923827 PMC5666308

[B12] Cui S. Erlichman J. Russo P. Haber B. A. Matthews R. P. (2011). Intrahepatic Biliary Anomalies in a Patient with Mowat-Wilson Syndrome Uncover a Role for the Zinc finger Homeobox Gene Zfhx1b in Vertebrate Biliary Development. J. Pediatr. Gastroenterol. Nutr. 52, 339–344. 10.1097/mpg.0b013e3181ff2e5b 21336163

[B13] Cui S. Leyva–Vega M. Tsai E. A. EauClaire S. F. Glessner J. T. Hakonarson H. (2013). Evidence from Human and Zebrafish that GPC1 Is a Biliary Atresia Susceptibility Gene. Gastroenterology 144, 1107–1115. 10.1053/j.gastro.2013.01.022 23336978 PMC3736559

[B14] Davit-Spraul A. Baussan C. Hermeziu B. Bernard O. Jacquemin E. (2008). CFC1 Gene Involvement in Biliary Atresia with Polysplenia Syndrome. J. Pediatr. Gastroenterol. Nutr. 46, 111–112. 10.1097/01.mpg.0000304465.60788.f4 18162845

[B111] Duriez M. Mandouri Y. Lekbaby B. Wang H. Schnuriger A. Redelsperger F. (2017). Alternative Splicing of Hepatitis B Virus: A Novel Virus/Host Interaction Altering Liver Immunity. J. Hepatol. 67, 687–699. 28600137 10.1016/j.jhep.2017.05.025 PMC6433284

[B15] del Río-Moreno M. Alors-Pérez E. González-Rubio S. Ferrín G. Reyes O. Rodríguez-Perálvarez M. (2019). Dysregulation of the Splicing Machinery Is Associated to the Development of Nonalcoholic Fatty Liver Disease. J. Clin. Endocrinol. Metab. 104, 3389–3402. 10.1210/jc.2019-00021 30901032 PMC6590982

[B16] Della Corte C. Mosca A. Vania A. Alterio A. Alisi A. Nobili V. (2016). Pediatric Liver Diseases: Current Challenges and Future Perspectives. Expert Rev. Gastroenterol. Hepatol. 10, 255–265. 10.1586/17474124.2016.1129274 26641319

[B17] Dong J. Feldmann G. Huang J. Wu S. Zhang N. Comerford S. A. (2007). Elucidation of a Universal Size-Control Mechanism in Drosophila and Mammals. Cell 130, 1120–1133. 10.1016/j.cell.2007.07.019 17889654 PMC2666353

[B18] Dongiovanni P. Meroni M. Mancina R. M. Baselli G. Rametta R. Pelusi S. (2018). Protein Phosphatase 1 Regulatory Subunit 3B Gene Variation Protects against Hepatic Fat Accumulation and Fibrosis in Individuals at High Risk of Nonalcoholic Fatty Liver Disease. Hepatol. Commun. 2, 666–675. 10.1002/hep4.1192 29881818 PMC5983109

[B19] Draijer L. Benninga M. Koot B. (2019). Pediatric NAFLD: an Overview and Recent Developments in Diagnostics and Treatment. Expert Rev. Gastroenterol. Hepatol. 13, 447–461. 10.1080/17474124.2019.1595589 30875479

[B20] Eichenmüller M. Trippel F. Kreuder M. Beck A. Schwarzmayr T. Häberle B. (2014). The Genomic Landscape of Hepatoblastoma and Their Progenies with HCC-like Features. J. Hepatol. 61, 1312–1320. 10.1016/j.jhep.2014.08.009 25135868

[B21] Elisofon S. A. Magee J. C. Ng V. L. Horslen S. P. Fioravanti V. Economides J. (2020). Society of Pediatric Liver Transplantation: Current Registry Status 2011-2018. Pediatr. Transpl. 24, e13605. 10.1111/petr.13605 31680409

[B22] Fawaz R. Baumann U. Ekong U. Fischler B. Hadzic N. Mack C. L. (2017). Guideline for the Evaluation of Cholestatic Jaundice in Infants: Joint Recommendations of the North American Society for Pediatric Gastroenterology, Hepatology, and Nutrition and the European Society for Pediatric Gastroenterology, Hepatology, and Nutrition. J. Pediatr. Gastroenterol. Nutr. 64, 154–168. 10.1097/mpg.0000000000001334 27429428

[B23] Feng J. Polychronidis G. Heger U. Frongia G. Mehrabi A. Hoffmann K. (2019). Incidence Trends and Survival Prediction of Hepatoblastoma in Children: a Population-Based Study. Cancer Commun. 39, 62. 10.1186/s40880-019-0411-7 PMC6813130 31651371

[B24] Gao E. Hercun J. Heller T. Vilarinho S. (2021). Undiagnosed Liver Diseases. Transl Gastroenterol. Hepatol. 6, 28. 10.21037/tgh.2020.04.04 33824932 PMC7829073

[B25] Garcia-Barceló M.-M. Yeung M.-Y. Miao X.-P. Tang C. S.-M. Chen G. So M.-T. (2010). Genome-wide Association Study Identifies a Susceptibility Locus for Biliary Atresia on 10q24.2. Hum. Mol. Genet. 19, 2917–2925. 10.1093/hmg/ddq196 20460270 PMC2893814

[B26] Gerhard G. S. Legendre C. Still C. D. Chu X. Petrick A. Distefano J. K. (2018). Transcriptomic Profiling of Obesity-Related Nonalcoholic Steatohepatitis Reveals a Core Set of Fibrosis-specific Genes. J. Endocr. Soc. 2, 710–726. 10.1210/js.2018-00122 29978150 PMC6018672

[B27] Girard M. Lacaille F. Verkarre V. Mategot R. Feldmann G. Grodet A. (2014). MYO5B and Bile Salt export Pump Contribute to Cholestatic Liver Disorder in Microvillous Inclusion Disease. Hepatology 60, 301–310. 10.1002/hep.26974 24375397

[B28] Girard M. Panasyuk G. (2019). Genetics in Biliary Atresia. Curr. Opin. Gastroenterol. 35, 73–81. 10.1097/mog.0000000000000509 30585837

[B29] Goldner D. Lavine J. E. (2020). Nonalcoholic Fatty Liver Disease in Children: Unique Considerations and Challenges. Gastroenterology 158, 1967–1983. 10.1053/j.gastro.2020.01.048 32201176

[B30] González-Mariscal L. Gallego-Gutiérrez H. González-González L. Hernández-Guzmán C. (2019). ZO-2 Is a Master Regulator of Gene Expression, Cell Proliferation, Cytoarchitecture, and Cell Size. Ijms 20, 4128. 10.3390/ijms20174128 PMC6747478 31450555

[B31] Götze T. Blessing H. Grillhösl C. Gerner P. Hoerning A. (2015). Neonatal Cholestasis - Differential Diagnoses, Current Diagnostic Procedures, and Treatment. Front. Pediatr. 3, 43. 10.3389/fped.2015.00043 26137452 PMC4470262

[B32] Gröbner S. N. Worst B. C. Worst B. C. Weischenfeldt J. Buchhalter I. Kleinheinz K. (2018). The Landscape of Genomic Alterations across Childhood Cancers. Nature 555, 321–327. 10.1038/nature25480 29489754

[B33] Henkel S. A. Squires J. H. Ayers M. Ganoza A. Mckiernan P. Squires J. E. (2019). Expanding Etiology of Progressive Familial Intrahepatic Cholestasis. Wjh 11, 450–463. 10.4254/wjh.v11.i5.450 31183005 PMC6547292

[B34] Hoang S. A. Oseini A. Feaver R. E. Cole B. K. Asgharpour A. Vincent R. (2019). Gene Expression Predicts Histological Severity and Reveals Distinct Molecular Profiles of Nonalcoholic Fatty Liver Disease. Sci. Rep. 9, 12541. 10.1038/s41598-019-48746-5 31467298 PMC6715650

[B35] Huang H. Shiffman M. L. Friedman S. Venkatesh R. Bzowej N. Abar O. T. (2007). A 7 Gene Signature Identifies the Risk of Developing Cirrhosis in Patients with Chronic Hepatitis C. Hepatology 46, 297–306. 10.1002/hep.21695 17461418

[B36] Jia D. Dong R. Jing Y. Xu D. Wang Q. Chen L. (2014). Exome Sequencing of Hepatoblastoma Reveals Novel Mutations and Cancer Genes in the Wnt Pathway and Ubiquitin Ligase Complex. Hepatology 60, 1686–1696. 10.1002/hep.27243 24912477

[B37] Jimenez M. Arechederra M. Ávila M. A. Berasain C. (2018). Splicing Alterations Contributing to Cancer Hallmarks in the Liver: central Role of Dedifferentiation and Genome Instability. Transl. Gastroenterol. Hepatol. 3, 84. 10.21037/tgh.2018.10.11 30505971 PMC6232053

[B38] Keitel V. Dröge C. Häussinger D. (2019). Targeting FXR in Cholestasis. Handb Exp. Pharmacol. 256, 299–324. 10.1007/164_2019_231 31201556

[B39] Khabou B. Siala-Sahnoun O. Gargouri L. Mkaouar-Rebai E. Keskes L. Hachicha M. (2016). In Silico investigation of the Impact of Synonymous Variants in ABCB4 Gene on mRNA Stability/structure, Splicing Accuracy and Codon Usage: Potential Contribution to PFIC3 Disease. Comput. Biol. Chem. 65, 103–109. 10.1016/j.compbiolchem.2016.10.008 27788395

[B40] Koch A. Denkhaus D. Albrecht S. Leuschner I. Von Schweinitz D. Pietsch T. (1999). Childhood Hepatoblastomas Frequently Carry a Mutated Degradation Targeting Box of the Beta-Catenin Gene. Cancer Res. 59, 269–273. 9927029

[B41] Koch A. Waha A. Hartmann W. Hrychyk A. Schüller U. Waha A. (2005). Elevated Expression of Wnt Antagonists Is a Common Event in Hepatoblastomas. Clin. Cancer Res. 11, 4295–4304. 10.1158/1078-0432.ccr-04-1162 15958610

[B42] Kopan R. Ilagan M. X. G. (2009). The Canonical Notch Signaling Pathway: Unfolding the Activation Mechanism. Cell 137, 216–233. 10.1016/j.cell.2009.03.045 19379690 PMC2827930

[B43] Kozlitina J. Smagris E. Stender S. Nordestgaard B. G. Zhou H. H. Tybjærg-Hansen A. (2014). Exome-wide Association Study Identifies a TM6SF2 Variant that Confers Susceptibility to Nonalcoholic Fatty Liver Disease. Nat. Genet. 46, 352–356. 10.1038/ng.2901 24531328 PMC3969786

[B44] Laquaglia M. J. Grijalva J. L. Mueller K. A. Perez-Atayde A. R. Kim H. B. Sadri-Vakili G. (2016). YAP Subcellular Localization and Hippo Pathway Transcriptome Analysis in Pediatric Hepatocellular Carcinoma. Sci. Rep. 6, 30238. 10.1038/srep30238 27605415 PMC5015017

[B45] Li J. Hua W. Ji C. Rui J. Zhao Y. Xie C. (2020). Effect of the Patatin-like Phospholipase Domain Containing 3 Gene (PNPLA3) I148M Polymorphism on the Risk and Severity of Nonalcoholic Fatty Liver Disease and Metabolic Syndromes: A Meta-Analysis of Paediatric and Adolescent Individuals. Pediatr. Obes. 15, e12615. 10.1111/ijpo.12615 32020770

[B46] Li L. Krantz I. D. Deng Y. Genin A. Banta A. B. Collins C. C. (1997). Alagille Syndrome Is Caused by Mutations in Human Jagged1, Which Encodes a Ligand for Notch1. Nat. Genet. 16, 243–251. 10.1038/ng0797-243 9207788

[B47] Lin W. Zhu C. Hong J. Zhao L. Jilg N. Fusco D. N. (2015). The Spliceosome Factor SART1 Exerts its Anti-HCV Action through mRNA Splicing. J. Hepatol. 62, 1024–1032. 10.1016/j.jhep.2014.11.038 25481564 PMC4404186

[B48] Lindsell C. E. Shawber C. J. Boulter J. Weinmaster G. (1995). Jagged: A Mammalian Ligand that Activates Notch1. Cell 80, 909–917. 10.1016/0092-8674(95)90294-5 7697721

[B49] Liu L. Y. Wang Z. L. Wang X. H. Zhu Q. R. Wang J. S. (2010). ABCB11 Gene Mutations in Chinese Children with Progressive Intrahepatic Cholestasis and Low Gamma Glutamyltransferase. Liver Int. 30, 809–815. 10.1111/j.1478-3231.2009.02112.x 19845854

[B50] Lovicu M. Lepori M. B. Incollu S. Dessì V. Zappu A. Iorio R. (2009). RNA Analysis of Consensus Sequence Splicing Mutations: Implications for the Diagnosis of Wilson Disease. Genet. Test. Mol. Biomarkers 13, 185–191. 10.1089/gtmb.2008.0089 19371217

[B51] Luo J.-H. Ren B. Keryanov S. Tseng G. C. Rao U. N. M. Monga S. P. (2006). Transcriptomic and Genomic Analysis of Human Hepatocellular Carcinomas and Hepatoblastomas. Hepatology 44, 1012–1024. 10.1002/hep.21328 17006932 PMC1769554

[B52] Makhmudi A. Supanji R. Putra B. P. Gunadi (2020). The Effect of APTR, Fn14 and CD133 Expressions on Liver Fibrosis in Biliary Atresia Patients. Pediatr. Surg. Int. 36, 75–79. 10.1007/s00383-019-04582-2 31549181

[B53] Mameli E. Lepori M. B. Chiappe F. Ranucci G. Di Dato F. Iorio R. (2015). Wilson's Disease Caused by Alternative Splicing and Alu Exonization Due to a Homozygous 3039-bp Deletion Spanning from Intron 1 to Exon 2 of the ATP7B Gene. Gene 569, 276–279. 10.1016/j.gene.2015.05.067 26031236

[B54] Mancina R. M. Dongiovanni P. Petta S. Pingitore P. Meroni M. Rametta R. (2016). The MBOAT7-TMC4 Variant Rs641738 Increases Risk of Nonalcoholic Fatty Liver Disease in Individuals of European Descent. Gastroenterology 150, 1219–1230. 10.1053/j.gastro.2016.01.032 26850495 PMC4844071

[B55] Mcdaniell R. Warthen D. M. Sanchez-Lara P. A. Pai A. Krantz I. D. Piccoli D. A. (2006). NOTCH2 Mutations Cause Alagille Syndrome, a Heterogeneous Disorder of the Notch Signaling Pathway. Am. J. Hum. Genet. 79, 169–173. 10.1086/505332 16773578 PMC1474136

[B56] Montes M. Sanford B. L. Comiskey D. F. Chandler D. S. (2019). RNA Splicing and Disease: Animal Models to Therapies. Trends Genet. 35, 68–87. 10.1016/j.tig.2018.10.002 30466729 PMC6339821

[B57] Nikeghbalian S. Malekhosseini S. A. Kazemi K. Arasteh P. Eghlimi H. Shamsaeefar A. (2021). The Largest Single Center Report on Pediatric Liver Transplantation. Ann. Surg. 273, e70–e72. 10.1097/sla.0000000000004047 32541224

[B58] Ningappa M. So J. Glessner J. Ashokkumar C. Ranganathan S. Min J. (2015). The Role of ARF6 in Biliary Atresia. PLoS One 10, e0138381. 10.1371/journal.pone.0138381 26379158 PMC4574480

[B59] Nobili V. Alisi A. Liu Z. Liang T. Crudele A. Raponi M. (2018). In a Pilot Study, Reduced Fatty Acid Desaturase 1 Function Was Associated with Nonalcoholic Fatty Liver Disease and Response to Treatment in Children. Pediatr. Res. 84, 696–703. 10.1038/s41390-018-0132-7 30120404 PMC6726123

[B60] Nobili V. Alisi A. Valenti L. Miele L. Feldstein A. E. Alkhouri N. (2019). NAFLD in Children: New Genes, New Diagnostic Modalities and New Drugs. Nat. Rev. Gastroenterol. Hepatol. 16, 517–530. 10.1038/s41575-019-0169-z 31278377

[B61] Overeem A. W. Li Q. Qiu Y. L. Cartón‐García F. Leng C. Klappe K. (2020). A Molecular Mechanism Underlying Genotype‐Specific Intrahepatic Cholestasis Resulting from MYO5B Mutations. Hepatology 72, 213–229. 10.1002/hep.31002 31750554 PMC7496772

[B62] Paris A. J. Snapir Z. Christopherson C. D. Kwok S. Y. Lee U. E. Ghiassi-Nejad Z. (2011). A Polymorphism that Delays Fibrosis in Hepatitis C Promotes Alternative Splicing of AZIN1, Reducing Fibrogenesis. Hepatology 54, 2198–2207. 10.1002/hep.24608 21837750 PMC3760215

[B63] Paulusma C. C. Folmer D. E. Ho-Mok K. S. de Waart D. R. Hilarius P. M. Verhoeven A. J. (2008). ATP8B1 Requires an Accessory Protein for Endoplasmic Reticulum Exit and Plasma Membrane Lipid Flippase Activity. Hepatology 47, 268–278. 10.1002/hep.21950 17948906

[B64] Petta S. Valenti L. Marra F. Grimaudo S. Tripodo C. Bugianesi E. (2016). MERTK Rs4374383 Polymorphism Affects the Severity of Fibrosis in Non-alcoholic Fatty Liver Disease. J. Hepatol. 64, 682–690. 10.1016/j.jhep.2015.10.016 26596542

[B65] Petta S. Valenti L. Tuttolomondo A. Dongiovanni P. Pipitone R. M. Cammà C. (2017). Interferon Lambda 4 Rs368234815 TT>δG Variant Is Associated with Liver Damage in Patients with Nonalcoholic Fatty Liver Disease. Hepatology 66, 1885–1893. 10.1002/hep.29395 28741298

[B66] Pietrobattista A. Veraldi S. Candusso M. Basso M. S. Liccardo D. Della Corte C. (2020). The Contribution of Plasma Oxysterols in the Challenging Diagnostic Work-Up of Infantile Cholestasis. Clinica Chim. Acta 507, 181–186. 10.1016/j.cca.2020.04.028 32353361

[B67] Pihlajamäki J. Lerin C. Itkonen P. Boes T. Floss T. Schroeder J. (2011). Expression of the Splicing Factor Gene SFRS10 Is Reduced in Human Obesity and Contributes to Enhanced Lipogenesis. Cel Metab. 14, 208–218. 10.1016/j.cmet.2011.06.007 PMC3167228 21803291

[B68] Qiu Y.-L. Gong J.-Y. Feng J.-Y. Wang R.-X. Han J. Liu T. (2017). Defects in Myosin VB Are Associated with a Spectrum of Previously Undiagnosed Low γ-glutamyltransferase Cholestasis. Hepatology 65, 1655–1669. 10.1002/hep.29020 28027573 PMC5413810

[B69] Rahman M. A. Krainer A. R. Abdel-Wahab O. (2020). SnapShot: Splicing Alterations in Cancer. Cell 180, 208. 10.1016/j.cell.2019.12.011 31951519 PMC7291876

[B70] Rajagopalan R. Tsai E. A. Grochowski C. M. Kelly S. M. Loomes K. M. Spinner N. B. (2020). Exome Sequencing in Individuals with Isolated Biliary Atresia. Sci. Rep. 10, 2709. 10.1038/s41598-020-59379-4 32066793 PMC7026070

[B71] Ramachandran P. Balamurali D. Peter J. J. Kumar M. M. Safwan M. Vij M. (2019). RNA-seq Reveals Outcome-specific Gene Expression of MMP7 and PCK1 in Biliary Atresia. Mol. Biol. Rep. 46, 5123–5130. 10.1007/s11033-019-04969-3 31342296

[B72] Sambrotta M. Strautnieks S. Strautnieks S. Papouli E. Rushton P. Clark B. E. (2014). Mutations in TJP2 Cause Progressive Cholestatic Liver Disease. Nat. Genet. 46, 326–328. 10.1038/ng.2918 24614073 PMC4061468

[B73] Sambrotta M. Thompson R. J. (2015). Mutations inTJP2, Encoding Zona Occludens 2, and Liver Disease. Tissue Barriers 3, e1026537. 10.1080/21688370.2015.1026537 26451340 PMC4574888

[B74] Sandahl T. D. Laursen T. L. Munk D. E. Vilstrup H. Weiss K. H. Ott P. (2020). The Prevalence of Wilson's Disease: An Update. Hepatology 71, 722–732. 10.1002/hep.30911 31449670

[B75] Santoro N. Zhang C. K. Zhao H. Pakstis A. J. Kim G. Kursawe R. (2012). Variant in the Glucokinase Regulatory Protein (GCKR) Gene Is Associated with Fatty Liver in Obese Children and Adolescents. Hepatology 55, 781–789. 10.1002/hep.24806 22105854 PMC3288435

[B76] Seeger C. Mason W. S. (2015). Molecular Biology of Hepatitis B Virus Infection. Virology 479-480, 672–686. 10.1016/j.virol.2015.02.031 25759099 PMC4424072

[B77] Sha Y. L. Liu S. Yan W. W. Dong B. (2019). Wnt/β-catenin Signaling as a Useful Therapeutic Target in Hepatoblastoma. Biosci. Rep. 39, BSR20192466. 10.1042/BSR20192466 31511432 PMC6757184

[B78] Shaalan U. F. Ibrahim N. L. Ehsan N. A. Sultan M. M. Naser G. M. Abd El-Fatah M. O. (2019). Reduced Immunohistochemical Expression of Hnf1β and FoxA2 in Liver Tissue Can Discriminate between Biliary Atresia and Other Causes of Neonatal Cholestasis. Appl. Immunohistochem. Mol. Morphol. 27, e32–e38. 10.1097/pai.0000000000000638 29406331

[B79] Shah A. B. Chernov I. Zhang H. T. Ross B. M. Das K. Lutsenko S. (1997). Identification and Analysis of Mutations in the Wilson Disease Gene (ATP7B): Population Frequencies, Genotype-Phenotype Correlation, and Functional Analyses. Am. J. Hum. Genet. 61, 317–328. 10.1086/514864 9311736 PMC1715895

[B80] Sharma A. Poddar U. Agnihotry S. Phadke S. R. Yachha S. K. Aggarwal R. (2018). Spectrum of Genomic Variations in Indian Patients with Progressive Familial Intrahepatic Cholestasis. BMC Gastroenterol. 18, 107. 10.1186/s12876-018-0835-6 29973134 PMC6032793

[B81] Sheldon R. D. Kanosky K. M. Wells K. D. Miles L. Perfield J. W. 2nd Xanthakos S. (2016). Transcriptomic Differences in Intra-abdominal Adipose Tissue in Extremely Obese Adolescents with Different Stages of NAFLD. Physiol. Genomics 48, 897–911. 10.1152/physiolgenomics.00020.2016 27764764 PMC5206389

[B82] Soussan P. Pol J. Garreau F. Schneider V. Pendeven C. L. Nalpas B. (2008). Expression of Defective Hepatitis B Virus Particles Derived from Singly Spliced RNA Is Related to Liver Disease. J. Infect. Dis. 198, 218–225. 10.1086/589623 18532883

[B83] Spector L. G. Birch J. (2012). The Epidemiology of Hepatoblastoma. Pediatr. Blood Cancer 59, 776–779. 10.1002/pbc.24215 22692949

[B84] Stenson P. D. Mort M. Ball E. V. Evans K. Hayden M. Heywood S. (2017). The Human Gene Mutation Database: towards a Comprehensive Repository of Inherited Mutation Data for Medical Research, Genetic Diagnosis and Next-Generation Sequencing Studies. Hum. Genet. 136, 665–677. 10.1007/s00439-017-1779-6 28349240 PMC5429360

[B85] Sumazin P. Chen Y. Treviño L. R. Sarabia S. F. Hampton O. A. Patel K. (2017). Genomic Analysis of Hepatoblastoma Identifies Distinct Molecular and Prognostic Subgroups. Hepatology 65, 104–121. 10.1002/hep.28888 27775819

[B86] Suzuki H. Kumar S. A. Shuai S. Diaz-Navarro A. Gutierrez-Fernandez A. De Antonellis P. (2019). Recurrent Noncoding U1 snRNA Mutations Drive Cryptic Splicing in SHH Medulloblastoma. Nature 574, 707–711. 10.1038/s41586-019-1650-0 31664194 PMC7141958

[B87] Suzuki T. Masui N. Kajino K. Saito I. Miyamura T. (1989). Detection and Mapping of Spliced RNA from a Human Hepatoma Cell Line Transfected with the Hepatitis B Virus Genome. Proc. Natl. Acad. Sci. 86, 8422–8426. 10.1073/pnas.86.21.8422 2554315 PMC298294

[B88] Takayasu H. Horie H. Hiyama E. Matsunaga T. Hayashi Y. Watanabe Y. (2001). Frequent Deletions and Mutations of the Beta-Catenin Gene Are Associated with Overexpression of Cyclin D1 and Fibronectin and Poorly Differentiated Histology in Childhood Hepatoblastoma. Clin. Cancer Res. 7, 901–908. 11309340

[B89] Terré S. Petit M. A. Bréchot C. (1991). Defective Hepatitis B Virus Particles Are Generated by Packaging and Reverse Transcription of Spliced Viral RNAs *In Vivo* . J. Virol. 65, 5539–5543. 10.1128/jvi.65.10.5539-5543.1991 1895403 PMC249055

[B90] Trobaugh-Lotrario A. D. Chaiyachati B. H. Meyers R. L. Häberle B. Tomlinson G. E. Katzenstein H. M. (2013). Outcomes for Patients with Congenital Hepatoblastoma. Pediatr. Blood Cancer 60, 1817–1825. 10.1002/pbc.24655 23798361

[B91] Tsai E. A. Grochowski C. M. Falsey A. M. Rajagopalan R. Wendel D. Devoto M. (2015). Heterozygous Deletion ofFOXA2Segregates with Disease in a Family with Heterotaxy, Panhypopituitarism, and Biliary Atresia. Hum. Mutat. 36, 631–637. 10.1002/humu.22786 25765999 PMC4477513

[B92] Tsai E. A. Grochowski C. M. Loomes K. M. Bessho K. Hakonarson H. Bezerra J. A. (2014). Replication of a GWAS Signal in a Caucasian Population Implicates ADD3 in Susceptibility to Biliary Atresia. Hum. Genet. 133, 235–243. 10.1007/s00439-013-1368-2 24104524 PMC3901047

[B93] Valenti L. Alisi A. Galmozzi E. Bartuli A. Del Menico B. Alterio A. (2010). I148M Patatin-like Phospholipase Domain-Containing 3 Gene Variant and Severity of Pediatric Nonalcoholic Fatty Liver Disease. Hepatology 52, 1274–1280. 10.1002/hep.23823 20648474

[B94] Van Der Woerd W. L. Mulder J. Pagani F. Beuers U. Houwen R. H. J. Van De Graaf S. F. J. (2015). Analysis of Aberrant Pre-messenger RNA Splicing Resulting from Mutations inATP8B1and Efficientin Vitrorescue by Adapted U1 Small Nuclear RNA. Hepatology 61, 1382–1391. 10.1002/hep.27620 25421123

[B95] van IJzendoorn S. C. D. Li Q. Qiu Y. l. Wang J. S. Overeem A. W. (2020). Unequal Effects of Myosin 5B Mutations in Liver and Intestine Determine the Clinical Presentation of Low‐Gamma‐Glutamyltransferase Cholestasis. Hepatology 72, 1461–1468. 10.1002/hep.31430 32583448 PMC7702107

[B96] Vitale G. Gitto S. Vukotic R. Raimondi F. Andreone P. (2019). Familial Intrahepatic Cholestasis: New and Wide Perspectives. Dig. Liver Dis. 51, 922–933. 10.1016/j.dld.2019.04.013 31105019

[B97] Vos T. Allen C. Arora M. Barber R. M. Bhutta Z. A. Brown A. (2016). Global, Regional, and National Incidence, Prevalence, and Years Lived with Disability for 310 Diseases and Injuries, 1990-2015: a Systematic Analysis for the Global Burden of Disease Study 2015. Lancet 388, 1545–1602. 10.1016/S0140-6736(16)31678-6 27733282 PMC5055577

[B98] Wang C. Zhou W. Huang Y. Yin H. Jin Y. Jia Z. (2018). Presumed Missense and Synonymous Mutations in ATP7B Gene Cause Exon Skipping in Wilson Disease. Liver Int. 38, 1504–1513. 10.1111/liv.13754 29637721

[B99] Wang H. Lekbaby B. Fares N. Augustin J. Attout T. Schnuriger A. (2019). Alteration of Splicing Factors' Expression during Liver Disease Progression: Impact on Hepatocellular Carcinoma Outcome. Hepatol. Int. 13, 454–467. 10.1007/s12072-019-09950-7 31140152

[B100] Ware S. M. Peng J. Zhu L. Fernbach S. Colicos S. Casey B. (2004). Identification and Functional Analysis of ZIC3 Mutations in Heterotaxy and Related Congenital Heart Defects. Am. J. Hum. Genet. 74, 93–105. 10.1086/380998 14681828 PMC1181916

[B101] Wattacheril J. Lavine J. E. Chalasani N. P. Guo X. Kwon S. Schwimmer J. (2017). Genome-Wide Associations Related to Hepatic Histology in Nonalcoholic Fatty Liver Disease in Hispanic Boys. J. Pediatr. 190, 100–107. 10.1016/j.jpeds.2017.08.004 28918882 PMC5690841

[B102] Webster N. J. G. (2017). Alternative RNA Splicing in the Pathogenesis of Liver Disease. Front. Endocrinol. 8, 133. 10.3389/fendo.2017.00133 PMC5478874 28680417

[B103] Wu H. L. Chen P. J. Tu S. J. Lin M. H. Lai M. Y. Chen D. S. (1991). Characterization and Genetic Analysis of Alternatively Spliced Transcripts of Hepatitis B Virus in Infected Human Liver Tissues and Transfected HepG2 Cells. J. Virol. 65, 1680–1686. 10.1128/jvi.65.4.1680-1686.1991 1705988 PMC239971

[B104] Wu P. Zhang M. Webster N. J. G. (2021). Alternative RNA Splicing in Fatty Liver Disease. Front. Endocrinol. 12, 613213. 10.3389/fendo.2021.613213 PMC7953061 33716968

[B105] Xiao J. Xia S.-y. Xia Y. Xia Q. Wang X.-r. (2014). Transcriptome Profiling of Biliary Atresia from New Born Infants by Deep Sequencing. Mol. Biol. Rep. 41, 8063–8069. 10.1007/s11033-014-3704-6 25192893

[B106] Xiao Y. Liu R. Li X. Gurley E. C. Hylemon P. B. Lu Y. (2019). Long Noncoding RNA H19 Contributes to Cholangiocyte Proliferation and Cholestatic Liver Fibrosis in Biliary Atresia. Hepatology 70, 1658–1673. 10.1002/hep.30698 31063660 PMC6819224

[B107] Yang Z.-X. Shen W. Sun H. (2010). Effects of Nuclear Receptor FXR on the Regulation of Liver Lipid Metabolism in Patients with Non-alcoholic Fatty Liver Disease. Hepatol. Int. 4, 741–748. 10.1007/s12072-010-9202-6 21286345 PMC2994619

[B108] Zappu A. Lepori M. B. Incollu S. Dessì V. Noli M. C. Mameli E. (2012). Feasibility of RNA Studies on Illegitimate Transcription for Molecular Characterization of Splicing Mutations in the ATP7B Gene: a Case Report. Mol. Cell Probes 26, 63–65. 10.1016/j.mcp.2011.10.002 22019423

[B109] Zhang J. Liu L. L. Gong J. Y. Hao C. Z. Qiu Y. L. Lu Y. (2020). TJP2 Hepatobiliary Disorders: Novel Variants and Clinical Diversity. Hum. Mutat. 41, 502–511. 10.1002/humu.23947 31696999

[B110] Zhu R. Baker S. S. Moylan C. A. Abdelmalek M. F. Guy C. D. Zamboni F. (2016). Systematic Transcriptome Analysis Reveals Elevated Expression of Alcohol-Metabolizing Genes in NAFLD Livers. J. Pathol. 238, 531–542. 10.1002/path.4650 26415102

